# Can Butterflies Evade Fire? Pupa Location and Heat Tolerance in Fire Prone Habitats of Florida

**DOI:** 10.1371/journal.pone.0126755

**Published:** 2015-05-27

**Authors:** Matthew D. Thom, Jaret C. Daniels, Leda N. Kobziar, Jonathan R. Colburn

**Affiliations:** 1 North Central Soil Conservation Research Lab, U.S. Department of Agriculture, Agricultural Research Service, Morris, Minnesota, United States of America; 2 Entomology and Nematology Department, University of Florida, Gainesville, Florida, United States of America; 3 Florida Museum of Natural History, Gainesville, Florida, United States of America; 4 School of Forest Resources and Conservation, University of Florida, Gainesville, Florida, United States of America; UMR INRA/INSA, BF2I, FRANCE

## Abstract

Butterflies such as the atala hairstreak, *Eumaeus atala* Poey, and the frosted elfin, *Callophrys irus* Godart, are restricted to frequently disturbed habitats where their larval host plants occur. Pupae of these butterflies are noted to reside at the base of host plants or in the leaf litter and soil, which may allow them to escape direct mortality by fire, a prominent disturbance in many areas they inhabit. The capacity of these species to cope with fire is a critical consideration for land management and conservation strategies in the locations where they are found. Survival of *E*. *atala* pupae in relation to temperature and duration of heat pulse was tested using controlled water bath experiments and a series of prescribed fire field experiments. Survival of *E*. *atala* pupae was correlated to peak temperature and heat exposure in both laboratory and field trials. In addition, *E*. *atala* survival following field trials was correlated to depth of burial; complete mortality was observed for pupae at the soil surface. Fifty percent of *E*. *atala* survived the heat generated by prescribed fire when experimentally placed at depths ≥ 1.75cm, suggesting that pupation of butterflies in the soil at depth can protect from fatal temperatures caused by fire. For a species such as *E*. *atala* that pupates above ground, a population reduction from a burn event is a significant loss, and so decreasing the impact of prescribed fire on populations is critical.

## Introduction

The disturbance caused by fire is highly influential in shaping, promoting, and sustaining certain successional stages, and is a major contributor to the dynamic nature of living systems [1]. The effects of fire are multifaceted in time, scale, and degree of severity. Fire may be temporarily destructive, by causing direct mortality of plants and animals in its path. Indirectly, the loss of food plants or other resources for animals at higher trophic levels post-fire can also have a negative effect [2,3]. However, fire can also promote ecosystem perpetuation, by releasing nutrients that were previously in unavailable forms such as dead wood, litter, and duff. The structural changes as a result of fire are also important in ecosystems where dominant species are dependent on bare mineral soil for seed germination, and for ruderal species that quickly occupy released growing space [4,5].

There are many examples of plants with traits that confer a higher probability of survival in fire prone areas than plants without those traits [6–8], but in animals such traits are not as common or as immediately apparent. Understanding how organisms cope with fire is critical to ensure effective management, especially for rare or imperiled organisms which exist in restricted and often isolated populations.

Prescribed fire is a land management tool used in a wide variety of ecosystems, and the changes in butterfly community and populations in response to this disturbance is well-studied. Butterfly species richness and abundance typically declines immediately following a controlled burn, and can recover over a period time unique to the particular species [9–12]. There is concern that remnant-dependent species that reside in small habitat patches that are repeatedly burned will become extinct, due to an inability for the population to recover sufficiently prior to additional disturbance [13–15].At the individual organism level, insects and other arthropods can survive fire by movement away from fire effects (e.g. smoke, heat) or by finding refuges [16,17]. Populations may be augmented by colonization of the burned area post-fire from surrounding unburned and occupied areas [9,18,19]. Both responses rely on suitable unburned areas within the range of the particular insect, either in the form of an escape refuge or as occupied habitat patches proximate to burned areas [20].

Enduring or evading the immediate effects of fire are alternative explanations. Although typically ascribed to plants, evaders are organisms where individuals may be partially destroyed by fire, but their genetic material remains viable and they reoccupy newly released habitat [21]. As applied to a particular species of butterfly, a particular life stage might be able to endure temperatures above the typical lethal range for other organisms due to physiological or behavioral adaptation. Burrowing into the soil to avoid disturbance is common in many vertebrates such as small rodents, turtles, and amphibians [22,23]. In the Lepidoptera, many different moth groups commonly pupate in the soil, such as saturniids, noctuids and sphingids [24–27]. Pupation in the soil is less well known for butterflies (the day-flying Lepidoptera including common families such as Hesperiidae, Nymphalidae, Pieridae, Papilionidae, and Lycaenidae), but escaping into the soil would likely provide some protection from fire for pupae. Fire temperatures are typically highest within the fuel being consumed, so residing below ground where ground (duff) fires are rare would likely result in a mitigation of heating [1,28,29].

Two at-risk species of related butterflies that can be found in Florida, the atala hairstreak, *Eumaeus atala* Poey, and the frosted elfin, *Callophrys irus* Godart, serve as examples of species that reside in fire-prone or managed habitats. The life history of *E*. *atala* includes close association with its sole larval host-plant, the cycad *Zamia integrifola* L., and pupation often occurs on the central cones or bases of fronds. *C*. *irus* also is closely associated with a single host plant, either *Lupinus perennis* L. or *Baptisia tinctoria* (L.) R.Br, and pupates in the leaf litter or soil at the base of the host plant. *Z*. *integrifola* and *L*. *perennis* are regarded as fire adapted, with *L*. *perennis* vigorously resprouting following fire, and producing higher biomass and a larger overall ground cover in frequently burned environments [30–34]. With their host plants demonstrating some adaptations to fire, it is likely that these butterfly species themselves are influenced by fire, yet how they survive these frequent disturbances has never been investigated.

The focus of this study was to explore factors and mechanisms to inform our understanding of butterfly fire tolerance strategy. We employed a coupled field and laboratory experimental approach using the atala hairstreak, *E*. *atala*, and observations of pupation depth of the frosted elfin, *C*. *irus*, for comparative purposes. Hypotheses tested include:(1) The depth of pupae in the litter, duff, and soil is correlated with post fire survival in *E*. *atala*, (2) heat from fire causes *E*. *atala* mortality, and as it increases in temperature and duration, so does the probability of mortality, and (3) the threshold temperature defining probable pupal mortality is not reached at the typical soil depths at which soil-dwelling butterfly pupae are found in frequently burned pine uplands. To test these hypotheses, several studies were successfully conducted including heat tolerance of *E*. *atala* butterfly pupae via laboratory experiments, *E*. *atala* pupal survival at different litter/soil depths following prescribed burning at the Ordway-Swisher Biological Station, Putnam County, Florida, and pupal depth measurements of the frosted elfin, *Callophrys irus*, *in situ* at Ralph E. Simmons Memorial State Forest, Nassau County, Florida.

## Methods

### Study Sites

Work was conducted at two sites in north Florida. Ralph E. Simmons Memorial State Forest (RESMSF) in Nassau County, Florida, contains an extant colony of *C*. *irus*, and was the study site for field observations of *C*. *irus* pupae location from August 2010 to June of 2012. *The C*. *irus* colony is restricted to an approximately 20 hectare section (lat 30.797°N, 81.949°W) in one of the sandhills, where the larval host-plant sundial lupine, *Lupinus perennis ssp*. *gracilis* (Nutt.) Dunn, is also located. The Ordway-Swisher Biological Station (OSBS) in Putnam County, Florida, served as the location for three experiments conducted on pupal survival following prescribed fire during July of 2012. OSBS is a 3,755 hectare year-round field station (lat 29.683°N, long 82°W) comprised of a mosaic of upland and wetland habitats. Both RESMSF and OSBS contain a diverse array of natural communities including sandhills, upland mixed forest, xeric hammocks, low pinelands, and a mixture of wetland habitats including riparian habitats along the St. Mary’s river (RESMSF) and numerous lakes (OSBS). Non-woody plants common to both sites include wiregrass, *Aristida stricta* Michx., gopherweed, *Baptisia lanceolata* (Walter) Elliot, wooly pawpaw, *Asimina incana* (W. Bartram) Exell, and pinewoods milkweed, *Asclepias humistrata* Walter. The woody plant community is characterized by longleaf pine *Pinus palustris* Mill., slash pine, *Pinus ellioti* Engelm., American persimmon, *Diospyros virginiana* L., turkey oak, *Quercus laevis* Walter, and various other *Quercus* sp. Soils at both sites consisted of moderately well drained fine sand.

RESMSF is managed for multiple uses including timber, hunting, and other public uses, with an emphasis on ecosystem management and ecological restoration of native communities. The prominent management technique used is prescribed fire, with dormant and growing season fires conducted in subdivided sandhill units approximately every other year since at least 2008 using a variety of techniques including aerial incendiary ignition and hand ignition by ground crews. All three experiments at OSBS were conducted in frequently burned (fire return intervals ranging from 1–3 years) sandhill pine and oak upland units, characterized by relatively open (fewer than 150 trees/ha) forests with grass and herbaceous dominants in the understory. Fires in this habitat are typically fast moving and carried by wiregrass, *A*. *stricta*, but varying amounts of leaf litter from *Q*. *laevis*, and pine needle litter from several pine species including *P*. *palustris*, also contribute to the fuel load. Historical land use in these units included both prescribed fire and wildfire, along with extensive grazing, during the 1900s. When the land was transferred to the University of Florida in 1980, active prescribed fire management was initiated. The OSBS management plan for this area has emphasized growing season prescribed fires for more than a decade.

Permits for work in RESMSF were approved by District Biologist Brian Camposano with the Florida Department of Agriculture and Consumer Services. Permits for field work at OSBS were approved by Steve Coates and the University of Florida.

### Study Organisms

Organisms used in this study include two at-risk Florida butterflies, the frosted elfin butterfly, *C*. *irus*, and a captive laboratory colony of the atala hairstreak, *E*. *atala*. Due to the numerous ecological and logistical challenges of using the univoltine frosted elfin butterfly in experiments, *E*. *atala* was instead used as the experimental subject; *E*. *atala* is also an at-risk species, but is more widely available and arguably less rare. *E*. *atala* shares a phylogenetic and ecological similarity to *C*. *irus*, is similar in size, shares an affinity to open understory forests where its larval host plant, *Zamia integrifola* L., is found, and produces continuous generations. *E*. *atala* stock was obtained from wild populations in Miami-Dade County in December, 2011, and from Broward, Palm Beach, and a different location in Miami-Dade County in May of 2012. Each cohort of *E*. *atala* consisted of approximately 200 final instar larvae and pupae gathered from occupied *Z*. *integrifolia* plants at the above locations. General colony rearing of *E*. *atala* occurred in an indoor lab at the Department of Entomology and Nematology at the University of Florida, Gainesville. This indoor area was maintained between 24–27°C and varied from 20–50% RH. In addition to nectar plants *Bidens alba* L., sweet almond bush, *Aloysia virgata* (Ruiz & Pav.) Pers., and scorpion tail *Heliotropium angiospermum* Murray, adults were offered small 15mL plastic centrifuge tube feeders adapted from previous designs that were filled with Gatorade [35,36].

General colony rearing of *E*. *atala* occurred in an indoor lab at the Department of Entomology and Nematology at the University of Florida, Gainesville. This indoor area was maintained between 24–27°C and varied from 20–50% RH. In addition to nectar plants *Bidens alba* L., sweet almond bush, *Aloysia virgata* (Ruiz & Pav.) Pers., and scorpion tail *Heliotropium angiospermum* Murray, adults were offered small 15mL plastic centrifuge tube feeders adapted from previous designs that were filled with Gatorade [35,36].

### Heat Tolerance of *E*. *atala* Pupae

A controlled lab experiment was conducted to test the tolerance of butterfly pupae to a range of combinations of increased temperature and duration, simulating heat exposure from fire while buried at depth. Tolerance to heat was measured as the survival of butterfly pupae to successful adult eclosion (emergence), hereafter referred to as survival. The range of possible temperatures and durations were determined from a search of published heat pulses in similar soil types such as sand or sandy loam [1,29]. General consensus of lethal temperature for animals is at about 50°C, so the range chosen for this experiment included this temperature plus an additional buffer beyond to give a firm end point. Lower temperatures were also of interest, as high durations of lower temperatures might also induce mortality. The resulting ranges tested included temperatures from 30°C to 65°C, and durations of 1 min to 55 min. Longer durations are certainly possible in some long-smoldering ground fires, but we chose 55 min as the maximum duration because the historically frequent fires in sandhill pine and oak forest were surface fires with relatively short residence times [37–40].

*E*. *atala* pupae were assigned a random temperature and duration combination from the listed ranges. Pupae themselves varied in age since pupation, a possible factor for successful survival to eclosion after exposure to a heat pulse. Pupae ages ranged from 5 to 15 days since pupation occurred, which was set as the point when the pupal molt was apparent and the larval head capsule was shed. Since all possible combinations of pupa age, duration, and temperature would result in an unmanageable experiment, pupae were lumped into general categories of early (5–7 days, n = 76), mid (8–11 days, n = 63) to late (12–15 days, n = 37) age and each range was assigned a similar assortment of temperature and duration combinations with the use of a random number generator (Table 1). This allowed for analysis of pupa age as a factor in survival to eclosion when exposed to a temperature and heat combination, as pupa maturity may lead to different survival probabilities.

**Table 1:** Experimental treatment setup for initial water bath experiment using *E*. *atala* pupae.

Peak Temperature (°C)	Duration (minutes)	Number of Pupae
27 (room temp.)	55	**Total = 14**
31–35	5–13	5
	14–27	9
	28–41	5
	42–55	4
		**Total = 23**
36–40	8–13	7
	14–27	5
	28–41	4
	42–55	5
		**Total = 21**
41–45	3–13	12
	14–27	7
	28–41	4
	42–55	5
		**Total = 28**
46–50	7–13	5
	14–27	10
	28–41	7
	42–55	5
		**Total = 27**
51–55	2–13	5
	14–27	7
	28–41	8
	42–55	2
		**Total = 22**
56–60	3–13	6
	14–27	2
	28–41	7
	42–55	8
		**Total = 23**
61–65	8–13	5
	14–27	3
	28–41	5
	42–55	5
		**Total = 18**

Each temperature and duration interval included pupae aged 5 to 15 days since pupation.

Experimental setup included placing individual pupae into glass test tubes that were loosely capped with a cotton ball, and each tube was labeled with the assigned temperature and duration combination. The lead of a T-type thermocouple was placed in contact with the pupa cuticle, and a data logger recorded the temperature reached over the course of each temperature and duration combination. At the end of the assigned duration, the pupae were promptly removed from the test tube and placed into a clear plastic cup with a lid, labeled with the age, temperature, and duration combination and set aside to be observed for eclosion. A small hole was opened in the lid, allowing the pupae to be misted with distilled water twice daily prior to eclosion. Also placed inside the plastic cup was a wooden stirring stick for the emerging butterfly to climb, aiding their movement to the underside of the cup lid. Viable adults were scored as those that were fully formed after eclosion from the pupa molt, and were capable of vertical flight as measured by release at a 1.5m height. Those that flew weakly to the ground or not at all were considered unviable, and were euthanized by freezing. Pupae were allowed 30 days to emerge, and after that were considered dead.

A follow up to this experiment was conducted to confirm that this experiment and its outcomes were repeatable. Taking the results from the first experiment, the temperature at which mortality first occurred was identified. The duration just short of causing mortality was selected as a treatment, as was the duration where morality occurred. This selection process was repeated for the highest temperature of any duration where some survival occurred. Two durations were chosen in the same manner as previously, just short of causing mortality and the duration where mortality first occurred. Finally two temperatures equally distributed between the high and low temperatures previously selected were chosen, again with a pair of durations on either side of the survival threshold. The result of the selection process was a total of eight unique temperature and duration treatments, to which a set of 6 differently aged *E*. *atala* pupae were each assigned (Table 2). A set of six pupae assigned as untreated controls were also handled and processed in the same manner as the heat treated pupae, only in a room temperature water bath (27°C). The experiment and follow-up handling of pupae and adults was conducted in exactly the same manner as in the first experiment.

**Table 2:** Experimental treatment setup for the follow-up water bath experiment using *E*. *atala* pupae.

Temperature (°C)	Duration (minutes)	
27 (room temp.)	53	
40	10	40
44	7	30
47	6	10
51	3	17

Each temperature and duration combination included a total of 6 pupae representing 6 ages since pupation: 2, 6, 7, 8, 11, and 15 days since pupation. Duration pairs for each temperature are for previously observed survival (low duration) and non-survival (high duration).

### Soil and Surface Temperatures and Post-Fire Survival of *E*. *atala*

In July of 2012, field experiments were conducted at OSBS to test the survival of *E*. *atala* pupae when exposed to prescribed burns. Two experiments on consecutive days (July 5^th^ and 6^th^) involved placement of live *E*. *atala* pupae in a management unit that was subsequently burned. A final experiment conducted on July 26^th^ was conducted in the same manner, minus placement of *E*. *atala* pupae, in order to increase the amount of temperature and treatment condition data that could be gathered. Within the management unit to be burned, a characteristic area in the interior was subjectively chosen to be the site where *E*. *atala* pupae were buried. Individual treatment sites were set up in such a way to account for small scale (1–3 m radius) heterogeneity in both high and low surface (litter and wiregrass) fuel loads, with sampling occurring within the range of fuel load variation present. Ocular estimates were used to quickly identify areas where litter and wiregrass cover was either patchy or continuous, as evidenced by the degree of exposed bare mineral soil, discontinuous wiregrass clumps, and sparse pine needle and oak leaf litter. After a specific area was chosen, the depth of burial was randomly assigned using a pen spun on a circle cut into six equal sections, each assigned a number ranging from the soil surface (0 cm) up to 5 cm depth, at 1 cm intervals.

Litter depth was measured prior to placement of *E*. *atala* pupae, as was general information on the litter and fuel types in the immediate area (~1 m radius) surrounding the treatment plot. If assigned the soil surface treatment, litter was temporarily displaced to arrange pupae at the soil surface, and then replaced in as close to the same arrangement as prior to removal. The temperature for surface treatments was recorded using a non-contact infrared thermometer (Raytek ST 20 Pro, Raytek Corporation) having an operating range of 23°C to 510°C +/- 1% or 1°C. The thermometer was aimed at a fixed point near each surface treatment in sequence every 30 seconds. Temperatures were recorded 15 minutes before the fire front entered the experiment area, during the passing of the fire front, and for approximately 15 minutes after passing of the flame front. The researcher taking the measurements was standing in the bed of a fire engine to ensure a clear line of sight to the measurement points. Fire ignition in each burn was conducted by ground crews using a drip torch, with the final burn also involving the use of two horseback mounted crew members carrying drip torches. Spotted strip head firing techniques were used to establish head fires (burning in the direction of the wind) so that fire would spread at a steady rate by the time the flaming front reached the research plots. Research plots were marked using surveying flags, to be visible by ground crews, and all research materials were removed post-fire.

Fire weather conditions were for the most part similar between experiments, though some differences were present. The July 5^th^ fire was a wind driven head fire (air temperatures at 2m: 33.7–34.0°C; wind direction and speed: W 3.5–3.7 km/hour; relative humidity: 50.8–52.8; rate of fire spread: 10 m/minute; estimated flame lengths of 1–2 m). The July 6^th^ fire was a slower moving backing fire (air temperatures at 2m: 31.4–32.0°C; wind direction and speed: W 3.8–4.5 km/hour; relative humidity: 61.0–64.0; rate of fire spread: 2.5 m/minute; estimated flame lengths of 1 m). Fire on July 26^th^ exhibited elements of both head and backing fires (air temperatures at 2m: 32.9–33.3°C; wind direction and speed: W 2.6–2.7 km/hour; relative humidity: 60.9–63.6; rate of fire spread: 2.5 m/minute; estimated flame lengths of 1 m). Both types of fire and the shift between each during a single burn are typical of prescribed burn behavior in this habitat, and so are representative of the conditions that would be experienced outside this experiment.

For pupae that were buried, the litter was treated in a manner similar to the surface treatments. Then, a small round hole was dug into the soil as close to the assigned depth as possible either by hand or using a garden bulb planter. Two pupae of the same age since pupation were placed at each cardinal direction, calibrated using a compass. In the center of this arrangement an iButton Thermocron Temperature Logger (DS1922L-F5, Maxim Integrated) was placed, which was programmed to begin logging approximately one hour prior to the passing of the fire front. The depth from the top of the iButton to the surface of the soil was measured and recorded, with the thickness of the iButton later added to the depth to get the actual depth. The treatments were covered back up with the removed soil plug, duff, and litter. Controls were set up in the exact same manner in a directly adjacent area that was not burned. A total of 8 pupae of 4 different ages were included in each depth treatment, with 6 treatments on July 5^th^, 9 treatments on July 6^th^ (Table 3).

**Table 3:** Experimental treatment numbers for prescribed fire survival experiments conducted at the Ordway-Swisher Biological Station, Putnam County, Florida, in July, 2012.

Experiment Date	Control (Unburned) Treatments	Experimental Treatments	Pupae Ages (days since pupal molt)	Experimental Treatment Depth (cm)	Total Pupae
7/5/2012	1	6	1, 9, 11, 16	0, 1.6, 1.9, 2.8, 3.8, 4.9	56
7/6/2012	2	9	2, 10, 12, 17	0, 0, 1.3, 1.5, 2.5, 2.9, 4.1, 4.5, 4.9	88
7/26/2012	3	19	N/A	1.1, 1.2, 1.4, 1.8, 1.9, 2.0, 2.2, 2.3, 2.6, 2.7, 2.8, 3.3, 3.4, 3.9, 4.1, 4.5, 5.0, 5.1, 5.6	N/A

Two *E*. *atala* pupae of four separate ages were buried in each treatment on the July 5^th^ and July 6^th^ experiments, for a total of eight pupae in each treatment. Experimental treatments included a single iButton temperature sensor buried in the soil if the assigned treatment depth >0 cm.

Approximately 20 min after the fire front passed, surface temperatures returned to similar values prior to the fire passing through the treatment area. Each experimental unit was then carefully excavated, with pupae individually removed and placed into clear plastic cups labeled with the pupal age, date, and treatment number. Pupae were transported back to the rearing lab, and monitored for survival in the following weeks. The designation of survival was performed in the same manner as in the controlled laboratory heat tolerance experiments (no malformations, positive vertical flight test). The iButton temperature sensors were also taken back to the lab for the uploading of temperature and time data.

### Pupal Depth Measurements of *C*. *irus*

**Fig 1:**
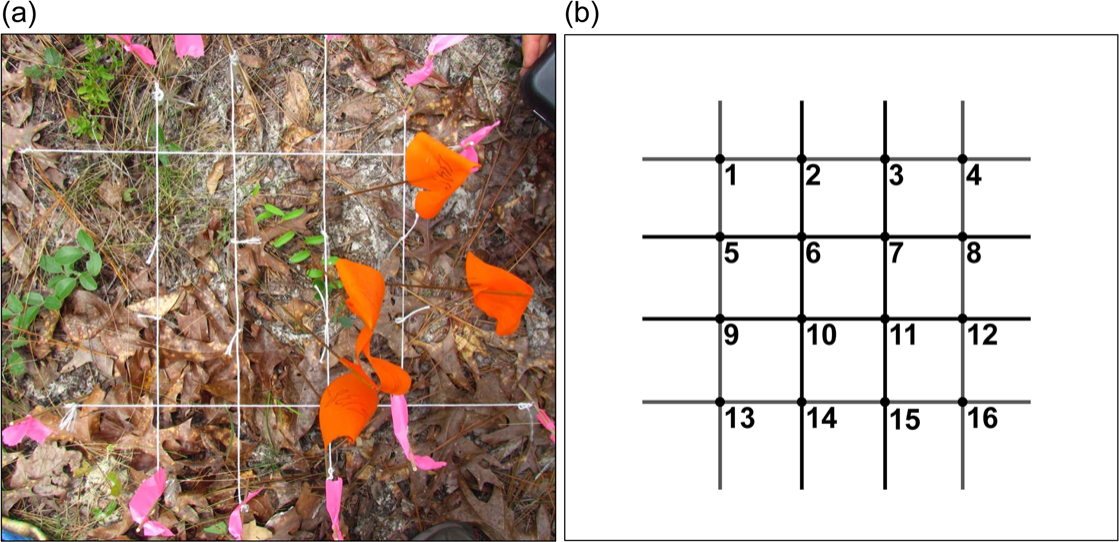
Datum setup and reference point diagram for field excavation of *C*. *irus* pupae at Ralph E. Simmons Memorial State Forest, Nassau County, Florida, May-June of 2012. (a) datum; (b) reference point diagram. Photo courtesy of M. Thom.

The *C*. *irus* colony at RESMSF was the location chosen for pupal depth excavation, a site where this species is known to persist with frequent fire. Previous work investigating pupation depth of insects has mostly focused on either laboratory or semi-field conditions, involving careful preparation of different types of soil in selected sites [24,25,41–43]. For the present study, *C*. *irus* pupae depth was investigated on August 31^st^, 2010, May 18^th^ to June 6^th^, 2011, and May 21st to June 5^th^, 2012, and utilized two methods for searching and excavation. The 2010 study involved simply searching the immediate area (1 m^2^) around a plant that supported late instar larvae documented during previous field observations, excavating in 1cm sections similar to [41]. The studies in 2011 and 2012 involved placing small enclosures around plants containing late instar larvae, to restrict their lateral movement during the larval wandering period, and aid in the actual location. Two types of enclosures were used: Live Monarch cloth mesh cages (Live Monarch Foundation) with the bottoms cut away and staked down around a focal plant (2011), and 30 cm sections of 20 cm diameter metal ducting inserted into the ground around a focal plant, with the top opening covered with tulle (2012). Temperature sensors (EasyLog USB) were also placed shaded and unshaded areas in and outside of the metal duct enclosures to monitor temperatures. Excavation in 2011 was conducted in the same manner as 2010. 2012 excavation utilized a datum, a technique borrowed from geological and archaeological excavation [44,45]. One can ensure precise measurements by measuring and recording the distance from the leveled datum to specific points such as the litter, duff, soil surface, and depth of objects (or pupae in this case) that are encountered, always referring to the level datum that was established prior to excavation. The datum setup used in this part of the study included setting a 24cm by 24cm square leveled datum centered on the focal plant that was caged with the metal ducting and tulle described earlier. The datum was created using straight wooden dowels upon which lengths of string were attached. At 6 cm intervals two lengths of string were also leveled across, forming one half of a grid, with each string marked at 6 cm intervals to form the final dimension, and making it a 16-point grid (Fig 1).

Prior to searching and excavation in all study years, the immediate area of the excavation site was described and photographed, including measuring litter and duff depths. Litter depth was measured by taking a dowel with a flat and level tip and placing it just in contact to the duff layer, marking off the depth on the dowel with a ruler or thumb. This spot was then measured to the nearest millimeter, and the whole process repeated for at least 5 other spots in a 40 cm radius. In the excavations involving the use of a datum, a total of 16 spots were measured equally across a grid (Fig 1B). Duff was measured in a similar way, by inserting a dowel into the duff until it reached the bare mineral soil surface. The litter was then carefully searched piece by piece for pupae, and set aside, as *C*. *irus* is known to pupate in either the litter or soil [46,47]. If no pupae were found, then the duff was searched in a manner identical to that of the litter, and also set aside. If no pupae were found in the duff layer, the soil was carefully excavated and searched to a depth of 10cm or until a pupa was uncovered. If a pupa was discovered, the depth was measured from the top of the soil surface to the pupa (n = 4), or using the nearest grid point to the level datum (n = 8). Excavation continued if more than one pupa was thought to be present (based on prior survey of number of mature larvae), but ceased if not. Pupae were replaced in the approximately same position, location, and depth they were found, and carefully covered with the corresponding amount of soil and leaf litter present before their excavation, to minimize any further disturbance.

### Data Analysis

Using the Expedata software (Sable Systems, Inc.), the temperature vs. time curves generated from both the thermocouple loggers and iButtons were imported and the integral was determined. For the temperature data from the lab study, the duration for the particular temperature experiment was highlighted and integrated. The integral value generated for all of the temperature data is essentially the cumulative heat that the pupae experienced, referred hereafter as “heat”. The baseline was set as the room temperature during the trials, and was 26 +/- 0.1°C for one thermocouple and logger, and 26.5 +/- 0.1°C for another that was used. Other possible factors such as peak temperature, time to peak temperature, temperature change, and the rate of temperature change were also extracted from the logged temperature file, and were used in regression analysis.

For the field burn experiments, finding the integral was slightly more complex because the baseline, the ambient temperature, was changing due to normal diurnal heating. To determine what actually was the pulse of heat that from the fire, beginning and end points were established. The beginning point was set as the moment right before a significant increase in temperature occurred over a short period of time, attributable to the passing of the flame front from the fire. Because this time point was slightly different for each treatment, it was determined from the rate of temperature change, calculated by dividing the current temperature for a reading by the previous reading, and subtracting one: *r = (T*_*n*_ *) / (T*_*n-1*_ *)* where *T*_*n*_ is the temperature at time *n* and *T*_*n-1*_ is the temperature at the previous time interval.

The beginning point was set where the rate was positive in 3 consecutive measurements. No such increase was observed in the unburned controls, and the beginning point also aligned precisely with a visual estimate of the beginning of heat pulse from the fire. The end points were set as the lowest temperature post-fire, right before heating from the diurnal flux begins again. This point was obtained by finding the halfway point between where cooling from the heat pulse levels off and diurnal heating begins again. This is the halfway point between the last negative rate change value and the first positive rate value in a series of 3 changes in a row that were positive, aligning with visual estimates of the end of the heat pulse.

The two-point drift-correction feature in the Expedata software was used to generate the baseline. Beginning and end points were selected in the program at the chosen time interval, as close to or slightly below the temperature recorded at that time. This was to ensure that when the line was connected it would not intersect or go above those points. What resulted was a baseline that sets the boundary between the heat pulse generated by the fire and the background diurnal temperature change. The integral was then taken over the interval of the heat pulse, with the newly generated baseline acting as zero.

Statistical analysis was conducted on sets of data collected from the initial lab water bath experiment and the field prescribed burning experiments. Logistic regression of survival of *E*. *atala* pupae as a function of the heat pulse recorded in each experiment was conducted in R using the separate data sets generated for lab and field experiments. Interactions between the heat pulse factors were included, and the regressions also contained pupal age as a predictive variable, and pupa weight for the lab experiment. A correlation matrix of the predictors was created for both experiments, and any terms highly correlated were modeled without the others present.

Model selection began by eliminating the most complex interaction terms one at a time, comparing it to the model with all the other similar level interaction terms. After they were determined to be singly non-significant, they were sequentially added to the model without that level of interaction terms. This process was repeated for all interaction levels and continued with removal of single terms. Models with all terms significant were compared to simpler models comparing the Akaike Information Criterion (AIC). As a rule of thumb, a simpler model (lesser number of terms) was chosen if the AIC did not decrease or increase by more than 2 when additional terms were added.

## Results

### Heat Pulse and Pupal Survival of *E*. *atala* During Prescribed Burns in Surrogate *C*. *irus* Habitat

**Fig 2:**
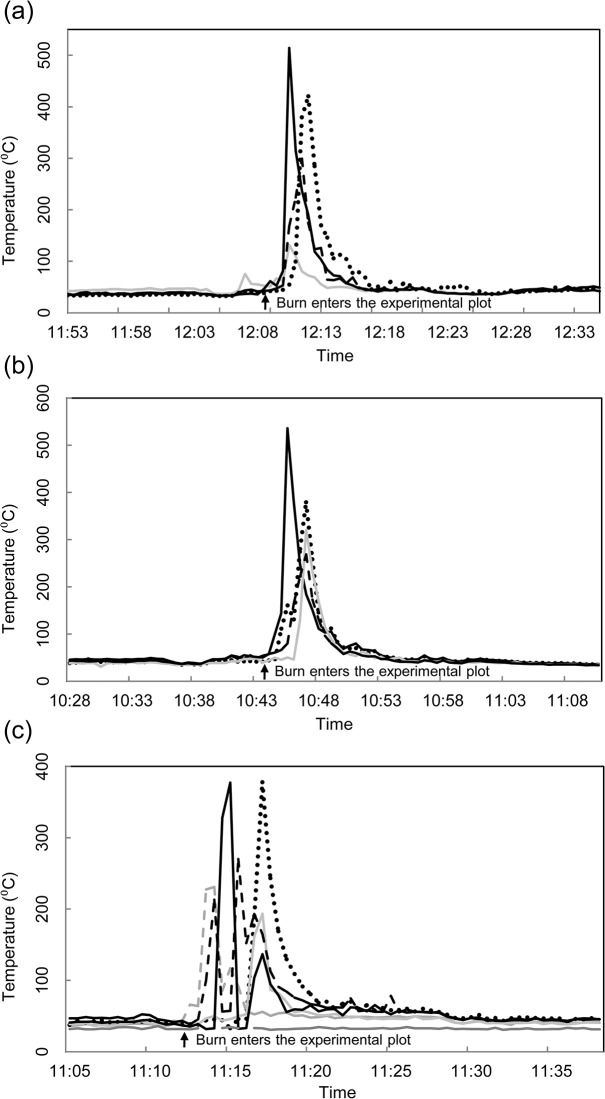
Soil surface temperatures as estimated using a non-contact infrared thermometer during controlled burning at the Ordway-Swisher Biological Station, Putnam County, Florida, July, 2012. (a) July 5^th^, 2012; (b) July 6^th^, 2012; (c) July 26^th^, 2012. Flat grey line in (c) is an unburned location monitored during the controlled burn.

Soil surface temperatures as measured by infrared thermometer for the July 5^th^ and 6^th^ prescribed burns at the OSBS exceeded 500°C (Fig 2), with lower temperature peaks recorded throughout the treatment plot (Fig 2A and 2B). Peak soil surface temperatures recorded for the July 26^th^ burn were approximately 375°C, again with considerable variation in peak temperature recorded in the treatment area (Fig 2C). An unburned position was also measured during the July 26^th^ burn, with no sharp rise in temperature recorded (Fig 2C, orange line). No *E*. *atala* pupae survived to adult eclosion in any of the soil surface treatments.

**Fig 3:**
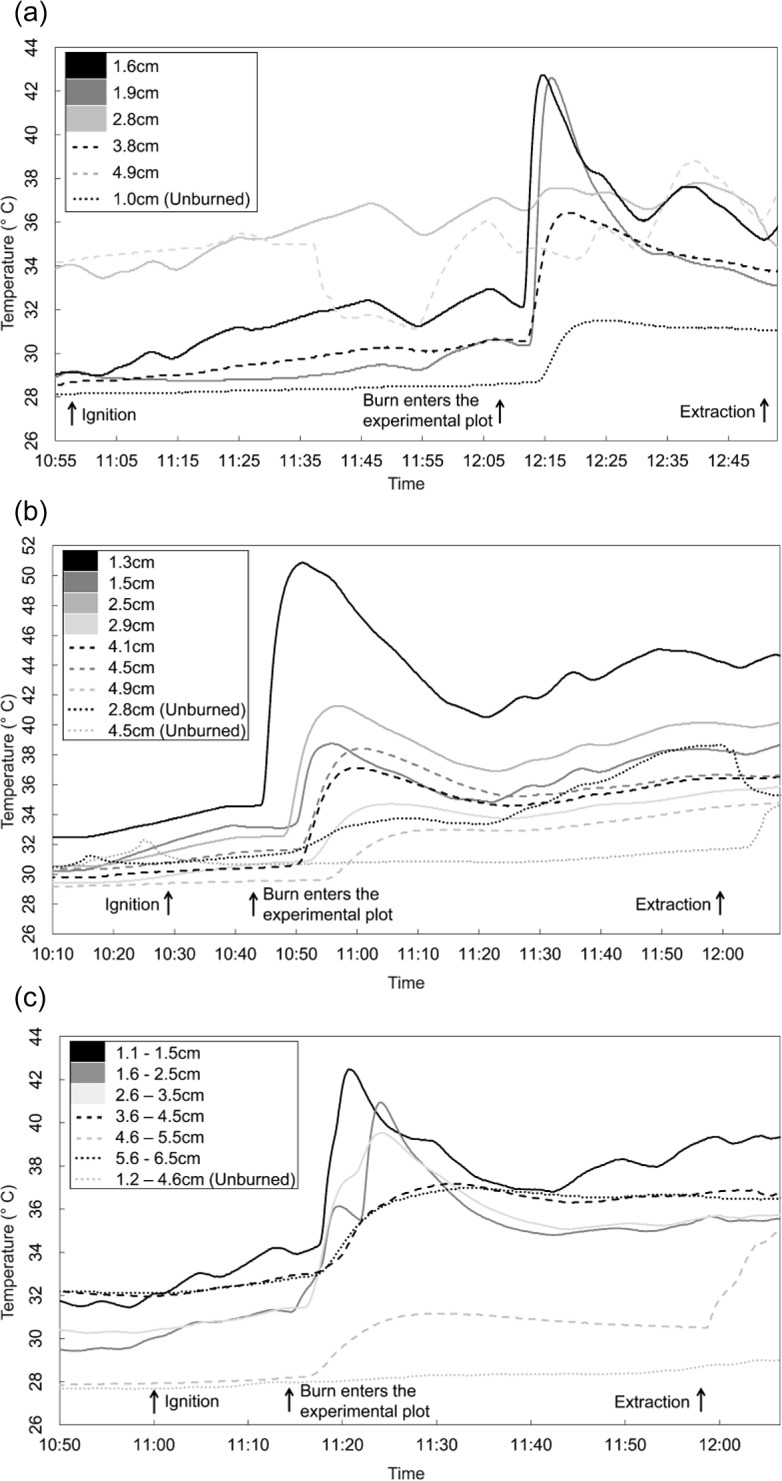
Soil temperatures as measured with iButton thermocrons during controlled burning at the Ordway-Swisher Biological Station, Putnam County, Florida, July 2012. (a) 5^th^, (b) 6^th^, (c) 26^th^, averaged soil temperatures.

Soil depth temperatures as measured by iButton sensors buried with *E*. *atala* pupae during the July 5^th^ burn peaked at 43°C in the two shallowest depths, 1.5cm and 1.9cm (Fig 3A). During the July 6^th^ burn, soil depth temperatures as recorded by iButton sensors buried with *E*. *atala* pupae peaked at approximately 51°C for the shallowest depth of 1.3cm (Fig 3B). Sensors buried in unburned sections recorded some heating, with the 2.8cm treatment recording displaying the most heating. Finally, averaged soil depth temperatures during the July 26^th^ burn recorded the highest peak temperatures in the shallowest treatments, peaking at 43°C at the 1.1–1.5cm range (Fig 3C). Sensors placed in the unburned area recorded little to no increase in temperature during the experiment.

**Fig 4:**
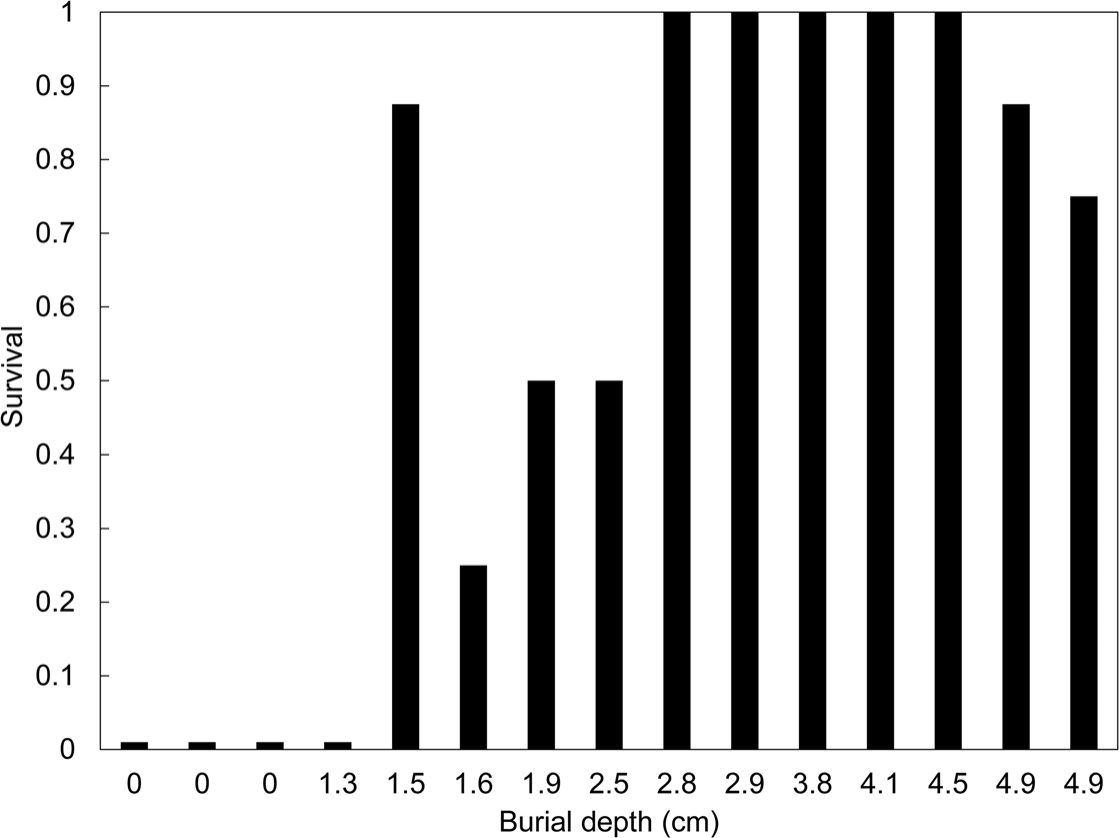
Proportion of *E*. *atala* pupae successfully eclosing following controlled burning on July 5^th^-6^th^ at the Ordway Swisher Biological Station, Putnam County, Florida. For each burial depth, n = 8 pupae.

Survival of *E*. *atala* pupae following the burial, prescribed burning, and removal is displayed in Fig 4. No adults survived for all three treatments on the soil surface or for those placed 1.3 centimeters below the surface. Survival was mixed for depths between 1.5 and 2.5cm, ranging from 25% to 88%. For burial depths of 2.8 to 4.1cm, 100% of *E*. *atala* survived, but there was a brief drop in survival for the final two depths of 4.9cm each (75–88%).

### Survival of *E*. *atala* Pupae Following Laboratory Water Bath Heat Treatment

**Fig 5:**
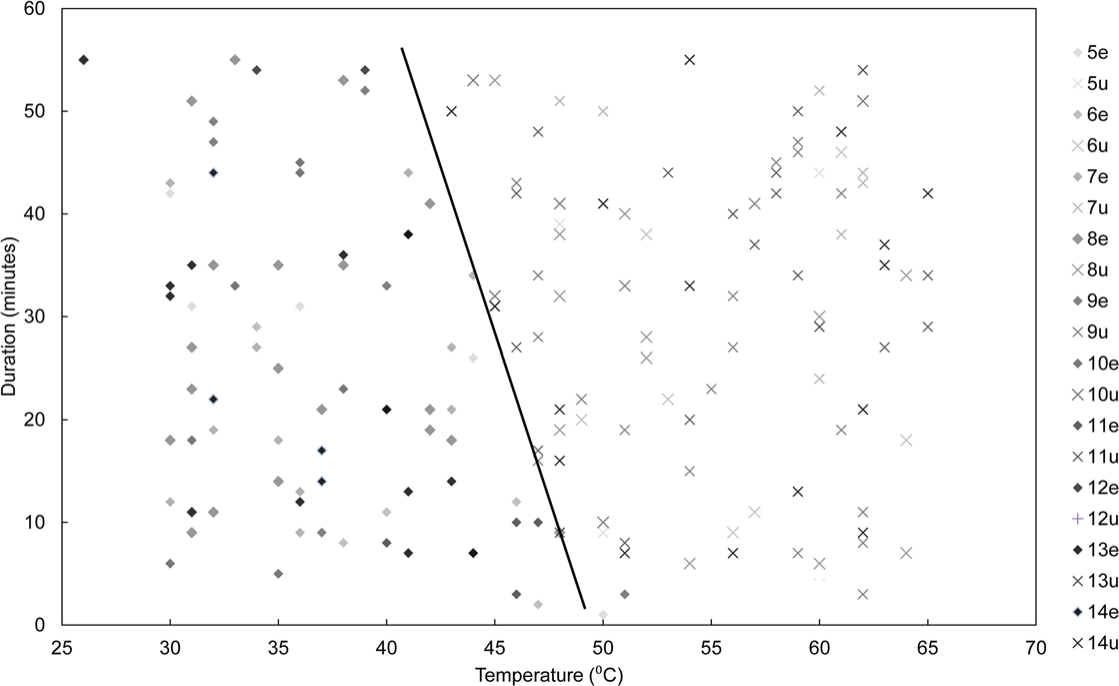
Plot of eclosion of *E*. *atala* following heated water bath treatment. ♦ = Successful adult eclosion, X = failure of adult to successfully eclose. Line represents a linear threshold between success and failure of *E*. *atala* to eclose into a viable adult following heated water bath treatment. Relative symbol darkness is related to age of pupae at the experiment (pupa age [eclosion status]).

A plot of *E*. *atala* survival following the initial water bath heating experiment is displayed in Fig 5. Survival varied by temperature and by duration, with a threshold between success and failure to successfully eclose that is roughly linear in nature. Failure began at about 40°C with a 50 min duration, and can be seen to roughly decrease linearly to 51°C with a 3 min duration.

*E*. *atala* survival from the follow-up water bath heating experiment was mostly consistent with the survival observed in the initial experiment. Survival of pupae in the 40°C treatment was high for both durations tested, 6/6 at 10 and 5/6 at 50 min. For the 44°C and 47°C treatments, some survival occurred in the long duration treatments, but much less so than for the short duration treatments: at 44°C, 6/6 survived the 7 min treatment vs 2/6 surviving the 30 min treatment; at 47°C 5/6 survived the 6 min treatment vs 2/6 for the 10 min treatment. Survival of the 51°C treatment was even more markedly different between durations: 5/6 for the 3 min treatment vs. 0/6 for the 17 min treatment.

### Factors Correlated to Survival of *E*. *atala* Pupae Following Prescribed Burning

**Fig 6:**
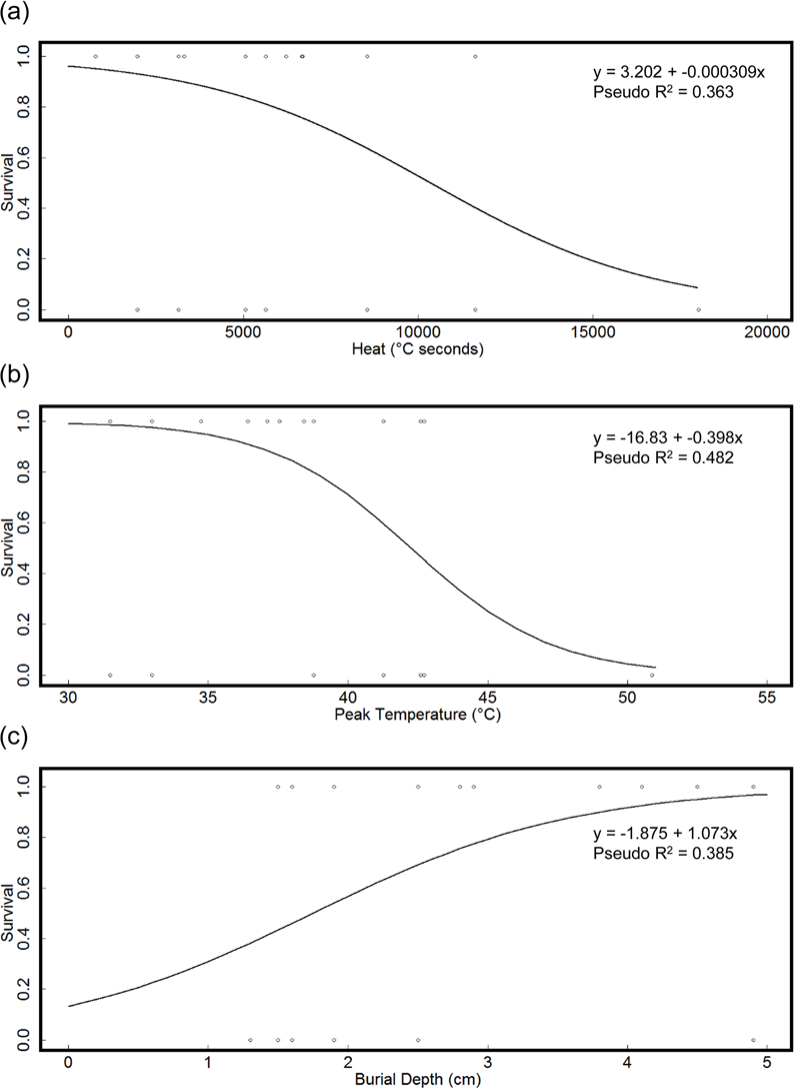
Probability of survival of *E*. *atala* pupa following controlled burning. Plot of survival probability of *E*. *atala* pupae to successful adult eclosion as a function of heat (a), peak temperature (b), and burial depth (c) from prescribed burning July 5-6^th^, 2012 at the Ordway Swisher Biological Station, Melrose, Florida. Nagelkurke/Cragg & Uhler’s pseudo R^2^ included as measure of model fit.

Heat, peak temperature, and burial depth are highly correlated, and so were analyzed in separate regression models. For all three models, pupa age was non-significant (p > 0.05) and is not displayed. Stepwise regression of the model including heat resulted in the most parsimonious and best fitting model being a single term model containing heat alone as a predictor (Table 4). The relationship is negative, with survival decreasing as heat increases (Fig 6A).

**Table 4:** Output summary of the logistic regression model of survival of *E*. *atala* pupae as a function of heat following prescribed burning July 5^th^-6th, 2012, at the Ordway Swisher Biological Station, Melrose, Florida.

	Estimate	Std. Error	z value	Pr (>|z|)	AIC	ΔAIC
Intercept	2.188	1.030	2.124	0.034	114.14	25.64
heat	-2.912e-04	7.425e-05	-3.922	8.76e-05	88.50	

ΔAIC included for the result of removal of significant model terms (p<0.05).

Stepwise regression of the model containing peak temperature resulted in the best fitting and most parsimonious model being a three factor function: peak temperature, time to peak temperature, and the interaction term peak temperature x time to peak temperature (Table 5). The direction of the correlations to survival were all negative (Fig 6B). No plots of survival versus “time to peak temperature” or “peak temperature x time to peak temperature” are included, as this result was not consistent across the regressions.

**Table 5:** Output summary of the logistic regression model of survival of *E*. *atala* pupae as a function of *in situ* peak temperature following prescribed burning July 5^th^-6th, 2012, at the Ordway Swisher Biological Station, Melrose, Florida.

	Estimate	Std. Error	z value	Pr (>|z|)	AIC	ΔAIC
Intercept	20.814	5.901	3.527	4.20e-04	114.14	36.77
peak temperature	-0.484	0.134	-3.613	3.03e-04	77.37	

ΔAIC included for the result of removal of significant model terms (p<0.05).

Stepwise regression of the model containing burial depth resulted in the best fitting and most parsimonious model being a three factor function including the terms burial depth, litter depth, and the interaction term burial depth x litter depth (Table 6). The relationship with burial depth is positive, with survival increasing as burial depth increases (Fig 6C).

**Table 6:** Output summary of the logistic regression model of survival of *E*. *atala* pupae as a function of burial depth, litter depth, and the interaction term burial depth*litter depth following prescribed burning July 5^th^-6th, 2012, at the Ordway Swisher Biological Station, Melrose, Florida.

	Estimate	Std. Error	z value	Pr (>|z|)	AIC	ΔAIC
Intercept	-4.579	1.408	-3.252	0.001	114.14	23.55
burial depth	1.897	0.500	3.796	1.47e-04	92.19	1.60
litter depth	0.094	0.039	2.427	0.015	93.25	2.66
burial depth: litter depth	-0.033	0.014	-2.404	0.016	90.59	

ΔAIC included for the result of removal of significant model terms (p<0.05).

### Factors Correlated to Survival of *E*. *atala* Pupae Following Water Bath Heating

**Fig 7:**
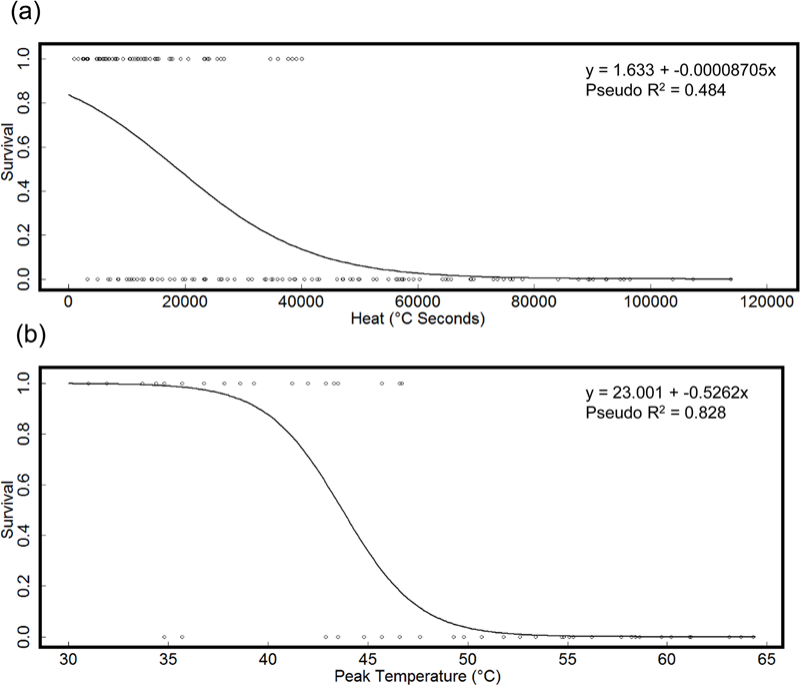
Probability of survival of *E*. *atala* pupae following water bath immersion. Plot of probability of survival of *E*. *atala* pupae to successful adult eclosion as a function of heat (A) and peak temperature (B) from the laboratory water bath experiment, February, 2012. Nagelkurke/Cragg & Uhler’s pseudo R^2^ included as measure of model fit.

Heat and peak temperature were highly correlated, and so were analyzed in separate models. For both models, pupa age and pupa weight were non-significant (p > 0.05), and are not displayed. Stepwise regression of the model including heat resulted in the most parsimonious and best fitting model to be a two factor model, heat and time to peak temperature as significant predictors of survival (Table 7). The direction of the correlation of survival to heat is negative, yet positive with time to peak temperature; survival decreases with increased heat, but increases with a longer time to peak temperature (Fig 7A). No plots of survival versus time to peak temperature are included, as this term was not consistent across the heat or peak temperature regressions.

**Table 7:** Output summary of the logistic regression model of survival of *E*. *atala* pupae as a function of heat and time to reach peak temperature following laboratory water bath experiment, February, 2012.

	Estimate	Std. Error	z value	Pr (>|z|)	AIC	ΔAIC
Intercept	0.0894	1.718	0.052	0.9585	216.6	69.08
heat	-1.08e-04	2.05e-05	-5.270	1.37e-07	147.52	2.58
time to peak temperature	0.102	0.048	2.119	0.034	144.94	

ΔAIC included for the result of removal of significant model terms (p<0.05).

Stepwise logistic regression of the model including peak temperature resulted in the best fitting and most parsimonious model to be a single factor model, peak temperature as the significant predictor of survival (Table 8). The correlation is negative, with survival decreasing as peak temperature increases (Fig 7B).

**Table 8:** Output summary of the logistic regression model of survival of *E*. *atala* pupae as a function of peak temperature following laboratory water bath experiment, February, 2012.

	Estimate	Std. Error	z value	Pr (>|z|)	AIC	ΔAIC
Intercept	24.700	5.624	4.393	1.12e-05	216.6	66.84
peak temperature	-0.551	0.109	-5.081	3.76e-07	66.84	

ΔAIC included for the result of removal of significant model terms (p<0.05).

### Regression Analysis Summary

Heat and peak temperature were consistent predictors of *E*. *atala* pupal survival. In both the laboratory water bath experiments and in the prescribed burn field experiments, logistic regression of the survival of *E*. *atala* pupae was significantly negatively correlated to heat (°C seconds) and peak temperature. The time taken to reach to peak temperature was also found to be a significant predictor in both of the heat only models of the lab and field experiments, but the nature of this relationship was different between experiments. Time to peak temperature in the lab experiment was shown to have a positive correlation, yet in the field experiment the opposite was true, with survival negatively correlated to time to reach peak temperature.

### Field Observations of Depth of Pupae of *C*. *irus*

From 2010 to 2012, a total of 39 excavations were undertaken to find *C*. *irus* pupae at Ralph E. Simmons Memorial State Forest, Nassau County, Florida, with a total of 12 pupae recovered. Of these, 8 of the pupae were located at the surface of the soil, in the duff and below the leaf litter. In general, leaf litter was composed primarily of turkey oak, sand oak, and persimmon leaves and small twigs, along with slash and longleaf pine needles. A lesser amount of grass blades contributed, with wiregrass being the most prominent. The following 4 pupae were found in the soil: 1 at 0.5cm, 2 at 2.0cm, and 1 at 3.0cm deep. The relatively low recovery rate of pupae could be attributed to a negative effect from the movement restriction devices. In a comparison of mean temperature in and outside the movement restricting devices, only 1 out of 4 comparisons showed significantly higher temperatures inside versus outside: mean temperature inside = 27.9°C, outside = 19.5°C (t = 7.7214, df = 320.27, p < 0.0001).

## Discussion

*E*. *atala* did not survive when placed in the leaf litter during the prescribed fire experiments, a position roughly analogous to their normal pupal location at the bases of fronds or cones of the larval host plant. Furthermore, the heat tolerance of *E*. *atala* in the water bath experiments did not exceed temperatures above 50°C, the typical thermal maximum for animals, and steadily decreased when duration of the heat pulse increased. This implies that *E*. *atala* does not evade fire by a physiological or behavioral trait. When placed in the soil, E. atala survival reached 50% at 1.75 cm, with greater survival at increasing depth. This corroborates previous findings in other fossorial insect groups such as the family Elateridae in the Coloptera [48,49]. Butterfly pupae such as those of *C*. *irus* at depths greater than 1.75 cm would have better chance of survival, but given the low number of *C*. *irus* found during pupa excavation (n = 12), it is difficult to speculate about whether this species is a fire evader. These results also come from a single population, one that has a history of frequent fire. A look at other populations of *C*. *irus*, including those with a range of fire regimes would be crucial to understanding whether or not it employs a strategy of fire evasion. However, it is compelling to note that the phenology of *C*. *irus* fits well with the typical fire timing: larvae pupate mid to late spring, and fires in Florida historically occur during late spring to early summer.

It is likely that there is a trade-off for enduring or escaping lethal temperatures. The upper thermal tolerance limit for animals is around 50°C, which should be noted as the upper limit of the core temperature; lower temperatures can also be lethal depending on the duration of the heat pulse [1,29]. There are examples of insects such as ants surviving temperatures that exceed 50°C, but typically involves only appendages and for short time periods [50,51]. High temperatures are physically demanding to organisms, as proteins denature and chemical reactions slow due to poorly functioning enzymes. There are biological pathways to resisting heat shock, but they can be costly [52,53].An immediate implication of this study is that it aids in better understanding the impact of prescribed fire management on litter and soil dwelling Lepidopteran pupae. Survival of *E*. *atala* was directly related to the amount of heat experienced in controlled laboratory experiments and in controlled burn situations. While not directly tested, mortality of egg and larvae residing on host plants would be extremely high in burned areas, as evidenced by temperatures exceeding 350°C at the soil surface in the three prescribed burn experiments, and the complete mortality of all *E*. *atala* pupae placed at the soil surface (Figs 2 and 4, respectively). Survival of fire by *C*. *irus* or other sandhill inhabiting organisms would therefore solely rest in their ability to avoid such temperatures, which for *C*. *irus* could only occur as a pupa that was buried in the soil to at least a couple of centimeters, corresponding to those times of the year when *C*. *irus* are pupae, approximately mid-May through early February the following year in Florida. Along with the proper seasonal timing of a prescribed fire, spatial extent is also highly important, as a fire that completely burns an entire area where *E*. *atala* are could result in the death of a large percentage of the entire pupa population. This suggests that the best way to manage an area containing a butterfly colony would be to only burn a portion of it each year, rotating burns in such a way that would not reduce the population, yet allowing for the positive benefits for host plant perpetuation to occur.

This conclusion, that spatial extent of prescribed fire is an important factor in the management of populations of *E*. *atala* or other insects like *C*. *irus*, falls in line with earlier work done in the Midwest in prairies and oak barrens communities. Specifically, non-burned areas can be important as refuge or source for populations of rare butterflies in small isolated habitat remnants, though higher abundance of *C*. *irus* was present in burned versus unburned habitat remnants [20]. It has also been shown that there is a greater species richness of other insects in burned versus unburned habitat remnants, emphasizing a need for the use of prescribed burning as a habitat management technique [10–12,54,55]. Further supporting this need are two explanations relevant to the current and latter work, the fire attrition and intermediate disturbance hypotheses. The fire attrition hypothesis states that burning at an inappropriate frequency results in a reduction in population sizes by not allowing sufficient recovery time, that too frequent of burns would reduce populations to become extinct [54]. Populations that are small and isolated would be at particularly greater risk. On the other hand, species such as *E*. *atala* and *C*. *irus* that are dependent on habitat and host plants maintained by relatively frequent disturbance also indirectly rely upon disturbance at some frequency. The intermediate disturbance hypothesis states that species richness is highest when disturbance is neither frequent nor too rare, and serves to fit with the ecology and life history of these two butterfly species [56,57]. Other rare butterfly species with narrow habitat requirements, such as the Fender’s blue, *Icaricia icarioides fenderi*, or the Karner blue, *Lycaeides melissa samuelis*, are also prone to this apparent dichotomy: reliance upon disturbance, yet an increased risk of extinction because of it [58–60]. This complexity can confound conservation, management and restoration efforts.

## Conclusions

Management suggestions resulting from the present study follow those from previous studies on rare insects (eg. [20,54,58]): divide the inhabited area into smaller units that are burned on a multi-year rotational schedule, to provide undisturbed refugia and support organisms across all life stages. Specifically, the units should be designated by the patches of larval host plants that are used by the given species, generated from multiyear surveys or monitoring efforts. The relative importance of managing for butterfly species such as *E*. *atala* or *C*. *irus* may be outweighed by other management objectives such as invasive species control, wildfire fuel load reduction, and other species specific management objectives. On the other hand, the small habitat areas that these butterflies inhabit in Florida or the Southern Coastal Plain could be managed in rotation with other methods potentially less damaging but are otherwise cost or time prohibitive in larger management units. Furthermore, a multi-year rotational burn cycle would aid in overall goals of fuel reduction and fit into a grander objective of greater biodiversity through increased habitat heterogeneity.
